# Direct Tumor Suppression by Anti-Basigin Antibody and Enhancement of Macrophage-Mediated Cancer Cell Phagocytosis under SIRPα Blockade

**DOI:** 10.24546/0100505582

**Published:** 2026-08-31

**Authors:** YIYAN YANG, TOMOKO TAKAI, NAOYA HATANO, YOJI MURATA, TAKASHI MATOZAKI, YOSHIMASA MANIWA, TAKENORI KOTANI

**Affiliations:** 1 Division of Thoracic Surgery, Department of Surgery, Kobe University Graduate School of Medicine, Kobe, Japan; 2 Division of Biosignal Regulation, Department of Biochemistry and Molecular Biology, Kobe University Graduate School of Medicine, Kobe, Japan; 3 Division of Molecular and Cellular Signaling, Department of Biochemistry and Molecular Biology, Kobe University Graduate School of Medicine, Kobe, Japan; 4 The Integrated Center for Mass Spectrometry, Kobe University Graduate School of Medicine, Kobe, Japan

**Keywords:** Basigin, SIRPα, CD47, Antitumor effect, Antibody-dependent cellular phagocytosis (ADCP)

## Abstract

The transmembrane protein CD47 expressed on tumor cells suppresses Fcγ receptor–mediated antibody-dependent cellular phagocytosis (ADCP) through its interaction with signal regulatory protein α (SIRPα), a transmembrane protein expressed on phagocytic cells such as macrophages. Although blockade of the CD47–SIRPα interaction enhances the efficacy of tumor-targeting antibodies such as rituximab, its antitumor activity remains limited in cancers lacking defined tumor-associated antigens. In this study, we identified the membrane protein Basigin and SIRPα as broadly applicable therapeutic targets for cancer. We found that the 8H4 antibody, originally identified in the course of other research, recognized mouse Basigin, suppressed proliferation of diverse cancer cell types, and induced cancer cell death. Treatment with the 8H4 antibody indeed inhibited tumor growth in vivo. Moreover, combined treatment with the 8H4 antibody and an anti-SIRPα antibody that blocks the CD47–SIRPα interaction markedly enhanced macrophage-mediated ADCP against diverse cancer cell types in vitro. These results suggest that targeting Basigin, particularly in combination with SIRPα blockade, may provide a basis for the development of macrophage-mediated immunotherapies applicable to cancers in which tumor-associated antigens have not yet been identified.

## INTRODUCTION

Cancer cells are known to actively regulate the tumor microenvironment (TME), which consists of surrounding cells such as immune cells, vascular endothelial cells, and stromal cells, to create conditions favorable for their own survival (1). Accordingly, intensive efforts have been made to develop therapeutic strategies that eliminate cancer cells by activating immune cells within the TME using antibodies and other agents. In particular, specific antibodies against molecules highly expressed on cancer cells, such as anti-CD20 antibody (rituximab) and anti-HER2 antibody (trastuzumab), have demonstrated remarkable antitumor effects by inducing antibody-dependent cellular phagocytosis (ADCP) or antibody-dependent cytotoxicity (ADCC) of immune effector cells against cancer cells (2–4). However, their efficacy is still limited to certain cancers. There are many cases where antibodies against molecules highly expressed in cancer do not show sufficient antitumor effects, and the reasons for this are not yet fully understood.

We have previously identified the CD47–signal regulatory protein α (SIRPα) signaling system, an intercellular signaling system, as one of the mechanisms by which cancer cells regulate the TME (5–7). CD47 is a ubiquitously expressed transmembrane protein with an extracellular immunoglobulin (Ig)-like domain and a short cytoplasmic tail (8). SIRPα is also a transmembrane protein, containing three extracellular Ig-like domains and tyrosine phosphorylation sites in its cytoplasmic region. SIRPα is predominantly expressed in macrophages, dendritic cells, and neurons (8). Studies focusing on hematopoietic cells have revealed that CD47 binds to the extracellular domain of SIRPα expressed on macrophages, thereby inhibiting Fcγ receptor–mediated ADCP (9). CD47 is overexpressed in various cancer cells, and previous studies suggest that cancer cells suppress their elimination by phagocytic cells such as macrophages through the expression of CD47 (10). We have previously demonstrated that anti-SIRPα antibodies and peptides that block the CD47–SIRPα interaction enhance macrophage-mediated ADCP of cancer cells and potentiate tumor elimination by therapeutic antibodies such as rituximab (5, 6).

However, for cancer types in which specific tumor-associated antigens have not been identified, it is difficult to induce antitumor effects by inhibiting the CD47–SIRPα signaling alone. In this study, we identified an antibody targeting Basigin, a membrane protein that is ubiquitously expressed across diverse cancer cells. In addition, we demonstrated that co-treatment with this antibody and an anti-SIRPα antibody synergistically enhanced ADCP activity against diverse cancer cell types.

## MATERIALS AND METHODS

### Animals

Wistar rats, C57BL/6J mice, and BALB/c mice were obtained from SLC (Hamamatsu, Japan). Systemic CD47-deficient (*Cd47*^−/−^) mice were generated as described previously (11). All animals were maintained at the Institute for Experimental Animals, Kobe University Graduate School of Medicine, under specific pathogen–free conditions. This study was approved by the Institutional Animal Care and Use Committee of Kobe University (Approval numbers: P201009-R4, P220812-R2, and P250912), and all animal experiments were performed according to Kobe University Animal Experimentation Regulations.

### Cell culture

The murine bladder cancer cell line MB49 was obtained from Merck Millipore (Burlington, MA). The murine colon cancer cell line MC38 was obtained from Kerafast (Boston, MA). The murine hepatoma cell MH134-TC was obtained from Institute of Development, Aging and Cancer, Tohoku University (Sendai, Japan). The murine Lewis lung carcinoma cell line LLC1 and the murine B-cell lymphoma cell line A20 were obtained from American Type Culture Collection (Manassas, VA). The murine glioma cell line GL261 was kindly provided by Dr. Sasayama at Kobe University. MB49, MC38, and LLC1 cells were cultured in Dulbecco’s modified Eagle’s medium (DMEM) (high glucose) (FUJIFILM Wako, Osaka, Japan) supplemented with 10% fetal bovine serum (FBS). GL261 cells were cultured in DMEM (low glucose) (FUJIFILM Wako) supplemented with 10% FBS. A20 cells were cultured in RPMI-1640 medium (FUJIFILM Wako) supplemented with 10% FBS. These cells were maintained at 37°C in a humidified incubator containing 5% CO_2_

### Antibodies and reagents

The information for the generation of the 4E8, 8H4, or 12D3 antibody is described below. A monoclonal antibody (mAb) to Flag (clone BM8) was from Sigma-Aldrich (Merck, Darmstadt, Germany). Allophycocyanin (APC)–conjugated mAb to F4/80 (clone BM8) was from BioLegend (San Diego, CA). Rabbit polyclonal antibodies to Caspase-3 and to cleaved Caspase-3 were from Cell Signaling Technology (Beverly, MA). Cy3–conjugated anti-rat IgG, Alexa Fluor 488–conjugated anti-rat or anti-mouse IgG, or Horseradish peroxidase–conjugated anti-rabbit IgG were from Jackson ImmunoResearch (West Grove, PA). 4′,6-diamidino-2-phenylindole (DAPI) was from Nacalai Tesque (Kyoto, Japan). Normal Rat IgG was from IBL (Fujioka, Japan) and normal Rat IgG2a was from Selleck Biotechnology (Yokohama, Japan). FITC-Annexinn V was from BioLegend. Carboxyfluorescein succinimidyl ester (CFSE) was from Sigma-Aldrich. Trypan blue solution and dimethyl sulfoxide (DMSO) were from FUJIFILM Wako.

### Generation of rat monoclonal antibodies against proteins on blood cells of *Cd47*^−/−^ mice and flow cytometry

For the generation of rat monoclonal antibodies, blood cells of *Cd47*^−/−^ mice were suspended in phosphate buffered saline (PBS) and mixed 1:1 with TiterMax Gold adjuvants (CytRx Corporation, Los Angeles, CA) to prepare a water-in-oil emulsion. For immunization, the emulsion was injected into the hind footpad of Wistar rats three times at 1-week intervals. Subsequently, lymphocytes were isolated from the popliteal lymph nodes and fused with P3U1 cells to generate hybridomas. Some hybridoma clones such as 4E8, 8H4, and 12D3 hybridoma were cultured in CELLine Disposable Bioreactor (CORNING, Corning, NY). The antibody-containing medium was harvested from the bioreactor, and each antibody was purified using a Protein G Sepharose column. The purification of the antibody was confirmed by SDS–PAGE, followed by Coomassie Brilliant Blue (CBB) staining of the gel. For flow cytometric analysis, 0.125 μL of peripheral blood from wild-type mice, or 1 × 10^5^ MB49, MC38, MH134-TC, or LLC1 cells were incubated with the 4E8, 8H4, or 12D3 antibody, or normal rat IgG (negative control), for 30 min on ice. Cells were then stained with an Alexa Fluor 488–labeled anti-rat IgG antibody as a secondary antibody. The stained cells were analyzed using a FACSVerse flow cytometer and FlowJo X software (BD Biosciences).

### Expression of FLAG-tagged mouse Basigin protein in HEK293 cells

To obtain mBasigin-FLAG/pcDNA3.1, Flag-tagged mouse Basigin DNA was amplified by PCR from a cDNA pool of RAW264.7 cells with the primers 5′-TTCAGAATTCCCACCATGGCGGCGGCGCTGCTGCTGG-3′ and 5′-AAACCTCGAGTCACTTGTCATCGTCGTCCTTGTAGTCGGTGGCGTTCCTCTGGCGTACA-3′. The resulting PCR product was subcloned into pcDNA3.1(+) (Thermo Fisher Scientific, Waltham, MA). To obtain mBasigin ΔD1-FLAG/pcDNA3.1, Flag-tagged mouse Basigin ΔD1 DNA, in which the coding sequence of the D1 domain was deleted, was amplified by PCR from mouse Basigin-FLAG/pcDNA3.1 using the primer pairs 5′-TTTTAAGCTTCCACCATGGCGGCGGCGCTGCTGCTGG-3′ and 5′-TTTTGAATTCCGCGCAGGCGCCTTGGCCGCTCA-3′, and 5′-TTTTGAATTCAAGGTCGGAAAGAAATCAGAGCAT-3′ and 5′-AAACCTCGAGTCACTTGTCATCGTCGTCCTTGTAGTCGGTGGCGTTCCTCTGGCGTACA-3′. The resulting two PCR products were subcloned into pcDNA3.1(+). To obtain mBasigin ΔD2-FLAG/pcDNA3.1, Flag-tagged mouse Basigin ΔD2 DNA, in which the coding sequence of the D2 domain was deleted, was amplified by PCR from mouse Basigin-FLAG/pcDNA3.1 using the primer pairs 5′-TTCAGAATTCCCACCATGGCGGCGGCGCTGCTGCTGG-3′ and 5′-TTTTGCGGCCGCCACATTGATCTCGCTTCTGCCCA-3′, and 5′-TTTTGCGGCCGCCATGGCAGCCCTCTGGCCCTTCCT-3′ and 5′-AAACCTCGAGTCACTTGTCATCGTCGTCCTTGTAGTCGGTGGCGTTCCTCTGGCGTACA-3′. The resulting two PCR products were subcloned into pcDNA3.1(+). The sequences of PCR products subcloned into pcDNA3.1(+) were verified by DNA sequencing (Eurofins Genomics, Tokyo, Japan). HEK293 cells were transfected with these expression vectors or pcDNA3.1(+) with the use of Lipofectamine 2000 (Thermo Fisher Scientific).

### Immunoprecipitation of the antigen recognized by the 8H4 antibody from red blood cell (RBC) ghosts of wild-type mice

RBC ghosts were prepared as shown previously (12), with minor modifications. In brief, peripheral blood was taken from wild-type mice and five volumes of 0.75% Dextran T-500 in 5 mM NaHPO_4_ (pH7.4) and 150 mM NaCl were added to the blood. They were sedimented for 30 minutes at 4°C. After aspiration of the supernatant, the Dextran sedimentation step was repeated. Then, cells were washed with PBS and were lysed by approximately 40 volumes of Mg^2+^ lysis buffer (5 mM NaHPO_4_ (pH7.4), 2 mM MgCl2, 1 mM dithiothreitol, and 20 μg/ml phenylmethylsulfonyl fluoride). After centrifugation, the supernatant was removed.

For preparation of the RBC ghost lysates, RBC ghosts were lysed with TNE buffer (20 mM Tris-HCl (pH7.5), 150 mM NaCl, 2 mM EDTA, 1% Nonidet P-40, 50 mM NaF, 1% protease inhibitor cocktail (Nacalai Tesque), and 5 mM 2-mercaptoethanol). The lysates were centrifuged at 17,500 × g for 15 min at 4°C, and the resulting supernatants were subjected to immunoprecipitation by the 8H4 antibody. Samples were subjected to SDS–PAGE, followed by silver staining using the Silver Stain II Kit Wako (FUJIFILM Wako).

### Mass spectrometry analysis

For identification of the antigen recognized by the 8H4 antibody, an approximately 45-kDa protein band was excised from a silver-stained gel onto which immunoprecipitated samples obtained using the 8H4 antibody from RBC ghost lysates had been applied. This silver-stained gel was performed by using the Silver Stain MS Kit (FUJIFILM Wako). In-gel digestion with trypsin was carried out according to a previously described protocol (13). Peptides were extracted with 0.1% formic acid and subsequently analyzed by LC–MS/MS using an LC-MS-IT-TOF instrument (Shimadzu, Kyoto, Japan) coupled to a nano reverse-phase LC system (Shimadzu) (14). MS/MS data were collected in the datum-dependent mode using LC-MS solution software (Shimadzu). The acquired spectra were processed with Mascot Distiller (Matrix Science, London, U.K.) to generate a single text file (containing the observed precursor peptide *m/z*, fragment ion *m/z*, and intensity values). Database searching was performed using Mascot (Matrix Science; version 2.3.01). Mascot searches were carried out with trypsin designated as the cleavage enzyme. Carbamidomethylation (Cys), oxidation (Met), and propionamide modification (Cys) were considered as variable modifications. Precursor and fragment mass tolerances were set at ±0.05 Da, allowing one missed cleavage. The SwissProt 2021_02 database was used for protein identification.

### Immunofluorescence analysis

Cells were fixed with 4% paraformaldehyde for 20 min at room temperature and stained with primary antibodies for 2 h at room temperature. They were then stained with fluorescent dye-labeled secondary antibodies as well as DAPI for 1 h at room temperature. Fluorescence images were obtained with a fluorescence microscope BX51 (Olympus, Tokyo, Japan).

### Effects of the 8H4 antibody on cancer cell proliferation and death

To assess the effects of the 8H4 antibody on cell proliferation and cell death, MC38, LLC1, MB49, GL261, and A20 cells were cultured in the presence of the 8H4 antibody or a control rat IgG2a antibody. Cell numbers were determined every 24 h after trypan blue staining. Trypan blue–negative cells were defined as viable cells, whereas trypan blue–positive cells were defined as dead cells. Antibodies were added to the culture medium at a final concentration of 10 μg/ml for MC38, 1 μg/ml for LLC1, 10 μg/ml for MB49, 10 μg/ml for GL261, and 10 μg/ml for A20 cells. Images were obtained with a microscope CKX53 (Olympus, Tokyo, Japan).

### Immunoblot analysis

MC38 cells were treated with 10 μg/ml normal rat IgG2a, 10 μg/ml 8H4 antibody or 20 μM Camptothecin for 24 hours. For immunoblot analysis, cells were lysed in RIPA buffer (20 mM Tris-HCl (pH7.5), 150 mM NaCl, 2 mM EDTA, 1% Nonidet P-40, 0.1% sodium deoxycholate, 0.1% sodium dodecyl sulfate, 50 mM NaF, 1% protease inhibitor cocktail (Nacalai Tesque), and 5 mM 2-mercaptoethanol). The lysates were centrifuged to remove insoluble debris, and the resulting supernatants were subjected to immunoblot analysis as previously described (15).

### Analysis of Annexin V-positive cells

MC38 cells were treated with 10 μg/ml normal rat IgG2a, 10 μg/ml 8H4 antibody or 20 μM Camptothecin for 24 hours. Cells were then stained with FITC Annexin V and DAPI in Annexin V binding buffer (Biolegend). The stained cells were analyzed using a MACS Quant 10 flow cytometer (Miltenyi Biotec, Bergisch Gladbach, Germany) and FlowJo X software (BD Biosciences).

### In vivo engraftment of cancer cells and intratumoral injection of the 8H4 antibody

A20 cells (5 × 10^6^ cells in 100 μl of PBS) were injected subcutaneously into the right flank of 6-week-old female BALB/c mice. Seven days after A20 cell injection, mice bearing tumors with a volume of ≥50 mm^3^ were injected intratumorally with either control rat IgG2a (50 μg, n = 15 mice) or the 8H4 antibody (50 μg, n = 15 mice) on days 7, 10, 14, and 17 after A20 cell injection. Tumor size was measured with the longest diameter (*a*) and the shortest diameter (*b*) with digital calipers, and tumor volume was calculated using the formula: *a* × *b*^2^/2.

### Preparation of mouse bone marrow derived macrophages (BMDMs)

Bone marrow–derived macrophages (BMDMs) of mice were prepared as described previously (5), with minor modifications. In brief, mouse bone marrow cells were harvested from the femurs and tibias, and red blood cells were lysed using ACK buffer (150 mM NH_4_Cl, 10 mM KHCO_3_). The bone marrow cells were then cultured in Iscove’s Modified Dulbecco’s Medium (IMDM) supplemented with 10% FBS and 10 ng/ml macrophage colony-stimulating factor (M-CSF) (PeproTech, Cranbury, NJ). These cells were cultured at 37°C in a humidified incubator containing 5% CO_2_. Non-adherent cells were removed during culture, and the resulting BMDMs were used for the phagocytosis assay from day 7 after seeding onward.

### Phagocytosis assay

BMDMs were transferred at a density of 1.5 × 10^4^ per well in 96-well plates and allowed to adhere overnight in IMDM supplemented with 10% FBS and 10 ng/ml M-CSF. The medium was then replaced with IMDM without FBS or M-CSF. After 2 h, 6 × 10^4^ CFSE-labeled cancer cells were added to each well, and incubated for 4 h in the absence or the presence of 20 μg/ml antibodies (20 μg/ml normal rat IgG2a; 10 μg/ml MY-1 antibody + 10 μg/ml normal rat IgG2a; 10 μg/ml 8H4 antibody + 10 μg/ml normal rat IgG2a; or 10 μg/ml MY-1 antibody + 10 μg/ml 8H4 antibody). The cells were then harvested, stained with an APC-conjugated mAb to F4/80, and analyzed using a MACS Quant 10 flow cytometer (Miltenyi Biotec) and FlowJo X software. Phagocytosis was calculated as the percentage of CFSE^+^ cells among F4/80^+^ cells (100 × F4/80^+^CFSE^+^ cells/total F4/80^+^ cells).

### Statistics

Data are presented as means ± SEM and were compared with one-way analysis of variance (ANOVA) or two-way repeated-measures ANOVA followed by Tukey’s multiple-comparison test. A *P* value of < 0.05 was considered statistically significant. All statistical analysis was performed with the use of GraphPad Prism 10.4.1 software (GraphPad, La Jolla, CA).

## RESULTS

### Production of the 8H4 antibody and its binding to various types of mouse cancer cell lines

The interaction of CD47 on RBCs with SIRPα on macrophages prevents Fcγ receptor–mediated phagocytosis of RBCs by macrophages (16). By contrast, the clearance of “unopsonized” *Cd47*^−/−^ RBCs from wild-type (WT) mice is markedly increased compared with WT RBCs (17). These previous reports suggest that CD47 on RBCs not only inhibits macrophage phagocytic activity but also likely masks the activity of an as yet unidentified molecule on RBCs that promotes their phagocytosis. To identify such a CD47-masked, as yet unidentified molecule on RBCs, we immunized rats with peripheral blood cells from *Cd47*^−/−^ mice to obtain monoclonal antibodies (mAbs) that block the phagocytosis by macrophages of *Cd47*^−/−^ RBCs.

We obtained multiple hybridoma clones that produced antibodies against a variety of proteins present in mouse blood cells. Figure 1A shows representative mouse blood cells stained with antibodies purified from the culture supernatants of individual hybridoma clones and analyzed by flow cytometry. The antibodies of hybridoma clones, which we named 4E8 and 8H4, well recognized wild-type blood cells, whereas those of the 12D3 hybridoma clone recognized only a small fraction of cells. Based on these results, the antibodies from 4E8 and 8H4 hybridomas were considered to recognize RBCs, which constitute the majority of cells in peripheral blood, whereas the antibody from 12D3 hybridoma was thought to recognize a minor population of cells present in peripheral blood. During the analysis of the antibodies of individual hybridomas, we noticed that the antibody from 8H4 hybridoma recognized various mouse cancer cell lines such as MB49 (a murine bladder cancer cell line), MC38 (a murine colon cancer cell line), MH134-TC (a murine hepatoma cell line), and LLC1 (a murine Lewis lung carcinoma line) cells (Figure 1B). We named the antibody produced by the 8H4 hybridoma as the 8H4 antibody. Using Rat Monoclonal Antibody Isotyping Test Kit (BIO-RAD, CA), we determined that the 8H4 antibody belongs to the rat IgG2a subclass and possesses a kappa light chain (Figure 1C). Immunostaining showed that the purified 8H4 antibody indeed stained MC38 cells (Figure 1D) and LLC1 cells (Figure 1E). Furthermore, the 8H4 antibody also recognized other cancer cell lines, including GL261 cells (a murine glioma cell line, Figure 1F) and A20 cells (a murine B-cell lymphoma cell line, Figure 1G). Given that the 8H4 antibody was an intriguing antibody capable of recognizing various types of mouse cancer cells, we therefore investigated this antibody as a separate line of study from our efforts to identify inhibitory antibodies targeting macrophage-mediated phagocytosis of *Cd47*^−/−^ RBCs.

### The antigen of the 8H4 antibody is the mouse Basigin protein

We sought to identify the antigen recognized by the 8H4 antibody. To purify the antigen, we performed immunoprecipitation using the 8H4 antibody and RBC ghost (RBCs that lack cytoplasmic content) lysates. We found that approximately 45-kDa proteins were immunoprecipitated by the 8H4 antibody, but not by normal rat IgG2a, from RBC ghost lysates (Figure 2A). In addition, these 45-kDa proteins were not detected when immunoprecipitation was performed with the 8H4 antibody and lysis buffer alone (without cell lysate) (Figure 2A). To identify the antigen recognized by the 8H4 antibody, the antigen was immunoprecipitated from RBC ghost lysates using the 8H4 antibody. An approximately 45-kDa protein band was excised from a silver-stained gel for mass spectrometry (Figure 2B), followed by in-gel digestion with trypsin and subsequent mass spectrometric analysis.

Mascot analysis identified mouse Basigin as a candidate protein among the approximately 45-kDa proteins immunoprecipitated by the 8H4 antibody. We indeed found that the 8H4 antibody recognized HEK293 cells expressing FLAG-tagged mouse Basigin protein, whereas it did not recognize HEK293 cells transfected with the empty vector (Figure 2C). Basigin, also known as CD147 or EMMPRIN, is a transmembrane glycoprotein belonging to the Ig superfamily. Basigin has isoforms known as Basigin-1, Basigin-2, Basigin-3, and Basigin-4 (18). Although the roles of Basigin-3 and Basigin-4 were not well understood, Basigin-1 and Basigin-2 have been well characterized (18–20). Basigin-1 contains three Ig domains—the D0 domain (I-set domain of Ig), the D1 domain (C2-set domain of Ig), and the D2 domain (I-set domain of Ig)—and is specifically expressed in the retina (21). Basigin-2 contains two Ig domains (D1 and D2 domains) and is considered to be the major isoform expressed in many cell types, including RBCs (19, 22). Immunostaining with the 8H4 antibody of HEK293 cells expressing FLAG-tagged mutant mouse Basigin proteins, each lacking a specific domain, demonstrated that this antibody recognized the D2 domain but not the D1 domain of the Basigin protein (Figure 2D). These results indicate that the 8H4 antibody targets mouse Basigin and specifically recognizes its D2 domain.

### Inhibition of cancer cell growth and survival by the 8H4 antibody

Given that Basigin has been implicated in cancer development (23), we investigated the effect of the 8H4 antibody on mouse cancer cell lines. To clarify the effects of the 8H4 antibody on cancer cells in vitro, we added

Similarly, the 8H4 antibody treatment suppressed the growth of LLC1 cells, MB49 cells, and A20 cells (Figure 3C, D, and F). In GL261 cells, although treatment with the 8H4 antibody showed a tendency to suppress cell proliferation, no statistically significant difference was observed compared with control IgG2a treatment. In addition, the 8H4 antibody treatment induced the cell death of LLC1, MB49, GL261, and A20 cells (Figure 3C–F). Immunoblot analysis showed that levels of cleaved Caspase-3, a marker of Caspase-3 activation and cellular apoptosis, were increased in MC38 cells following treatment with the 8H4 antibody (Figure 3G). Treatment with the 8H4 antibody also increased the proportion of Annexin V–positive MC38 cells, indicating that the antibody enhanced apoptosis (Figure 3H). These results suggest that the 8H4 antibody induces apoptosis, at least in MC38 cells, and may exert similar effects in other mouse cancer cell lines.

Next, we evaluated the antitumor activity of the 8H4 antibody in vivo using a murine subcutaneous tumor transplantation model. A20 cells were subcutaneously inoculated into mice, and on day 7 after transplantation (when the tumor size reached ≥50 mm^2^), antibodies were administered intratumorally. Compared with tumors treated with a control antibody (normal rat IgG2a), tumors treated with the 8H4 antibody showed marked regression (Figure 4). These results suggest that the 8H4 antibody has an antitumor activity in vivo in the A20 tumor model. Furthermore, these findings support Basigin as a potential therapeutic target for cancer treatment.

### Impact of combination with the 8H4 antibody and an anti-SIRP**α** antibody (MY-1 antibody) on phagocytosis of cancer cells by macrophages

The combination of an anti-SIRPα antibody, which blocks the CD47–SIRPα interaction, and other antitumor antibody, which opsonizes tumor cells by recognizing the tumor-associated antigen, strongly promotes phagocytosis of tumor cells by macrophages (7). Indeed, the combination therapy with an anti-SIRPα antibody and other antitumor antibody such as rituximab suppresses tumor growth in vivo (5, 24). On the other hand, an antitumor antibody is disadvantageous in that it cannot exert effects on tumor cells that do not express its antigen. Given that the 8H4 antibody recognized various mouse cancer cell lines, we examined whether the 8H4 antibody could enhance macrophage-mediated phagocytosis of cancer cells in vitro. When normal rat IgG2a was added to the culture medium together with the 8H4 antibody or an anti-SIRPα antibody (MY-1), BMDM-mediated phagocytosis of MC38 cells was not strongly enhanced. In contrast, the combination of the 8H4 antibody with the MY-1 antibody promoted BMDM-mediated phagocytosis of MC38 cells more strongly than the combination with normal rat IgG2a (Figure 5A and B). Similarly, the combination of the 8H4 antibody and the MY-1 antibody markedly enhanced BMDM-mediated phagocytosis of MB49 cells, GL261 cells and A20 cells (Figure 5D–F). Although treatment with the combination of these antibodies tended to enhance BMDM-mediated phagocytosis of LLC1 cells, no statistically significant enhancement of phagocytosis was observed in LLC1 cells (Figure 5C). Collectively, the combination of the 8H4 antibody and the MY-1 antibody enhanced macrophage-mediated ADCP in several cancer cell lines.

## DISCUSSION

Cancer therapies that eliminate tumor cells by activating immune cells using antitumor agents are being actively developed. However, the therapeutic efficacy of such approaches remains limited to a subset of cancer types. Even antibodies targeting molecules that are highly expressed on tumor cells often fail to exert sufficient antitumor activity. In this study, we demonstrated that the 8H4 antibody, an anti-mouse Basigin antibody, exhibits antitumor effects such as inhibition of cell proliferation and induction of cell death across a wide range of cancer cell types. Importantly, the 8H4 antibody was also shown to have antitumor activity in vivo. Moreover, we showed that the combination of the 8H4 antibody with an anti-SIRPα antibody that blocks CD47–SIRPα signaling markedly enhances macrophage-mediated ADCP against diverse cancer cell types.

The 8H4 antibody obtained in this study is unique in that it was not obtained through target-directed antibody development with a predetermined target molecule, was instead serendipitously discovered during an unbiased immunization approach using the cells themselves. Specifically, we immunized rats with blood cells from *Cd47*^−/−^ mice to obtain antibodies that recognize antigens on *Cd47*^−/−^ RBCs. We obtained hybridoma clones that produced antibodies that can recognize mouse blood cells. Among these, we focused on the 8H4 antibody, produced by hybridoma clone 8H4, because the 8H4 antibody recognized various mouse cancer cell lines.

We identified the antigen of the 8H4 antibody as mouse Basigin by mass spectrometry analysis of the antigen biochemically purified from mouse RBC membranes using the 8H4 antibody. Basigin is a membrane protein known to regulate various cellular functions by binding to various molecules. This protein is highly expressed in many types of tumors such as glioblastoma, lung cancer, and colorectal carcinoma, and it has been considered a promising target for cancer therapy (18, 25). In cancer cells, Basigin has been shown to regulate cell proliferation, apoptosis, metabolism, adhesion, migration, invasion, and metastasis, and Basigin-mediated signaling generally promotes cancer malignancy (23, 25). In the present study, we demonstrated that the 8H4 antibody inhibits proliferation of various cancer cell lines while inducing cell death. We also demonstrated that the 8H4 antibody suppresses A20 tumor growth in vivo. Furthermore, analyses using MC38 cells suggested that the 8H4 antibody induces apoptosis in cancer cells. These findings suggest that the 8H4 antibody has the potential to exert antitumor effects against various types of cancer by inhibiting proliferation and inducing cell death such as apoptosis. Basigin proteins are known to interact with various molecules; for example, the D1 domain interacts with cyclophilin A/B, and the D2 domain interacts with monocarboxylate transporters (MCT1/MCT4) and integrins, thereby inducing a variety of intracellular signals such as PI3K, MAPK, and NF-known to regulate various cellular functions by binding to various molecules. This protein is highly expressed in many types of tumors such as glioblastoma, lung cancer, and colorectal carcinoma, and it has been considered a promising target for cancer therapy (18, 25). In cancer cells, Basigin has been shown to regulate cell proliferation, apoptosis, metabolism, adhesion, migration, invasion, and metastasis, and Basigin-mediated signaling generally promotes cancer malignancy (23, 25). In the present study, we demonstrated that the 8H4 antibody inhibits proliferation of various cancer cell lines while inducing cell death. We also demonstrated that the 8H4 antibody suppresses A20 tumor growth in vivo. Furthermore, analyses using MC38 cells suggested that the 8H4 antibody induces apoptosis in cancer cells. These findings suggest that the 8H4 antibody has the potential to exert antitumor effects against various types of cancer by inhibiting proliferation and inducing cell death such as apoptosis. Basigin proteins are known to interact with various molecules; for example, the D1 domain interacts with cyclophilin A/B, and the D2 domain interacts with monocarboxylate transporters (MCT1/MCT4) and integrins, thereby inducing a variety of intracellular signals such as PI3K, MAPK, and NF-known to regulate various cellular functions by binding to various molecules. This protein is highly expressed in many types of tumors such as glioblastoma, lung cancer, and colorectal carcinoma, and it has been considered a promising target for cancer therapy (18, 25). In cancer cells, Basigin has been shown to regulate cell proliferation, apoptosis, metabolism, adhesion, migration, invasion, and metastasis, and Basigin-mediated signaling generally promotes cancer malignancy (23, 25). In the present study, we demonstrated that the 8H4 antibody inhibits proliferation of various cancer cell lines while inducing cell death. We also demonstrated that the 8H4 antibody suppresses A20 tumor growth in vivo. Furthermore, analyses using MC38 cells suggested that the 8H4 antibody induces apoptosis in cancer cells. These findings suggest that the 8H4 antibody has the potential to exert antitumor effects against various types of cancer by inhibiting proliferation and inducing cell death such as apoptosis. Basigin proteins are known to interact with various molecules; for example, the D1 domain interacts with cyclophilin A/B, and the D2 domain interacts with monocarboxylate transporters (MCT1/MCT4) and integrins, thereby inducing a variety of intracellular signals such as PI3K, MAPK, and NF-

One of the major findings of this study is that the combined use of the 8H4 antibody and anti-SIRPα antibody enhanced the ADCP activity of macrophages against various cancer cells in vitro. We have previously shown that combining anti-SIRPα antibodies with rituximab synergistically enhances ADCP activity in macrophages and antitumor efficacy (5, 24). However, no agents that should be used in combination with anti-SIRPα antibodies have been identified for cancer types for which specific tumor-associated antigens have not been identified. We demonstrated that the 8H4 antibody, when used in combination with an anti-SIRPα antibody, markedly enhanced the phagocytic activity of macrophages against various types of cancer cell lines in vitro, indicating the possibility that the combination of the 8H4 antibody and anti-SIRPα antibody has antitumor activity. The lack of significant enhancement in LLC1 cells suggests that additional factors, such as differences in antigen expression or susceptibility to macrophage-mediated phagocytosis, may influence the efficacy of this combination; nevertheless, Basigin and SIRPα may represent promising therapeutic targets for cancers lacking well-defined tumor-associated antigens. Accordingly, the combined use of anti-Basigin and anti-SIRPα antibodies warrants further investigation as a potential strategy for cancer treatment. However, this study was based primarily on in vitro and ex vivo analyses. Although we also evaluated the antitumor effect of the combination treatment in vivo, no statistically significant improvement over either 8H4 monotherapy or anti-SIRPα monotherapy was observed under the current experimental conditions. Because both monotherapies exhibited substantial antitumor activity in this model, a ceiling effect may have limited our ability to detect an additional benefit of the combination treatment. Therefore, the enhanced therapeutic potential of combining 8H4 with anti-SIRPα antibody is currently supported mainly by the increased ADCP activity observed in vitro. Further studies will be required to determine whether this enhanced ADCP activity translates into superior antitumor efficacy in vivo and to define the experimental conditions under which synergistic effects can be detected.

In summary, we identified an anti-Basigin antibody, 8H4, that possesses both direct and immune-mediated antitumor activity. The findings of this study provide a conceptual and experimental basis for the development of macrophage-centered immunotherapy applicable to various cancer types, including rare cancers and cancers of unknown primary origin. Establishing in vivo proof of concept and elucidating its mechanism of action will be an important step toward clinical application. Future studies should evaluate the efficacy and safety of the combination therapy in multiple tumor models, establish strategies for systemic administration while minimizing adverse effects, clarify Fc-dependent versus signaling-mediated mechanisms, and assess potential toxicity related to Basigin expression in normal tissues and blood cells.

## Figures and Tables

**Figure 1:**
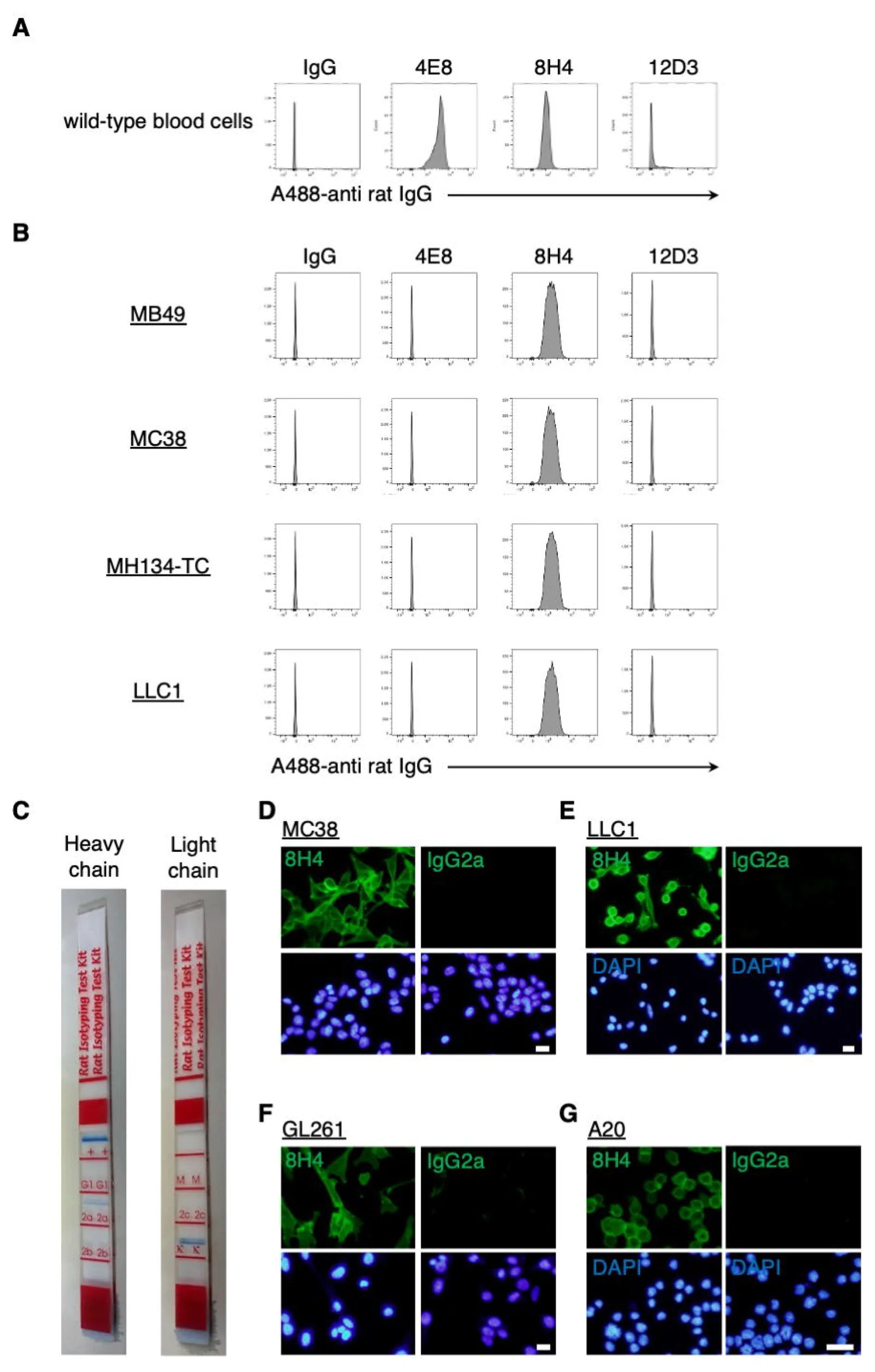
Production of the 8H4 antibody and its binding to various types of mouse cancer cell lines (A) Representative flow cytometric analysis of wild-type mouse red blood cells immunostained with the 4E8, 8H4, or 12D3 antibody. Normal Rat IgG (IgG) is used as a negative-control. Alexa Fluor 488–labeled anti-rat IgG (A488-anti rat IgG) antibody is used as a secondary antibody. (B) Representative flow cytometric analysis of mouse cancer cell lines (MB49, MC38, MH134-TC, or LLC1 cells) immunostained with the 4E8, 8H4, or 12D3 antibody. Normal Rat IgG (IgG) is used as a negative-control. (C) Isotyping of the 8H4 antibody. (D–G) Representative immunostaining of MC38 (D), LLC1 (E), GL261 (F), or A20 cells (G) with (upper left panel, 8H4) or without the 8H4 antibody (upper right panel, IgG2a) are shown. Nuclei are counterstained with DAPI (lower panels). Scale bars, 20 μm.

**Figure 2:**
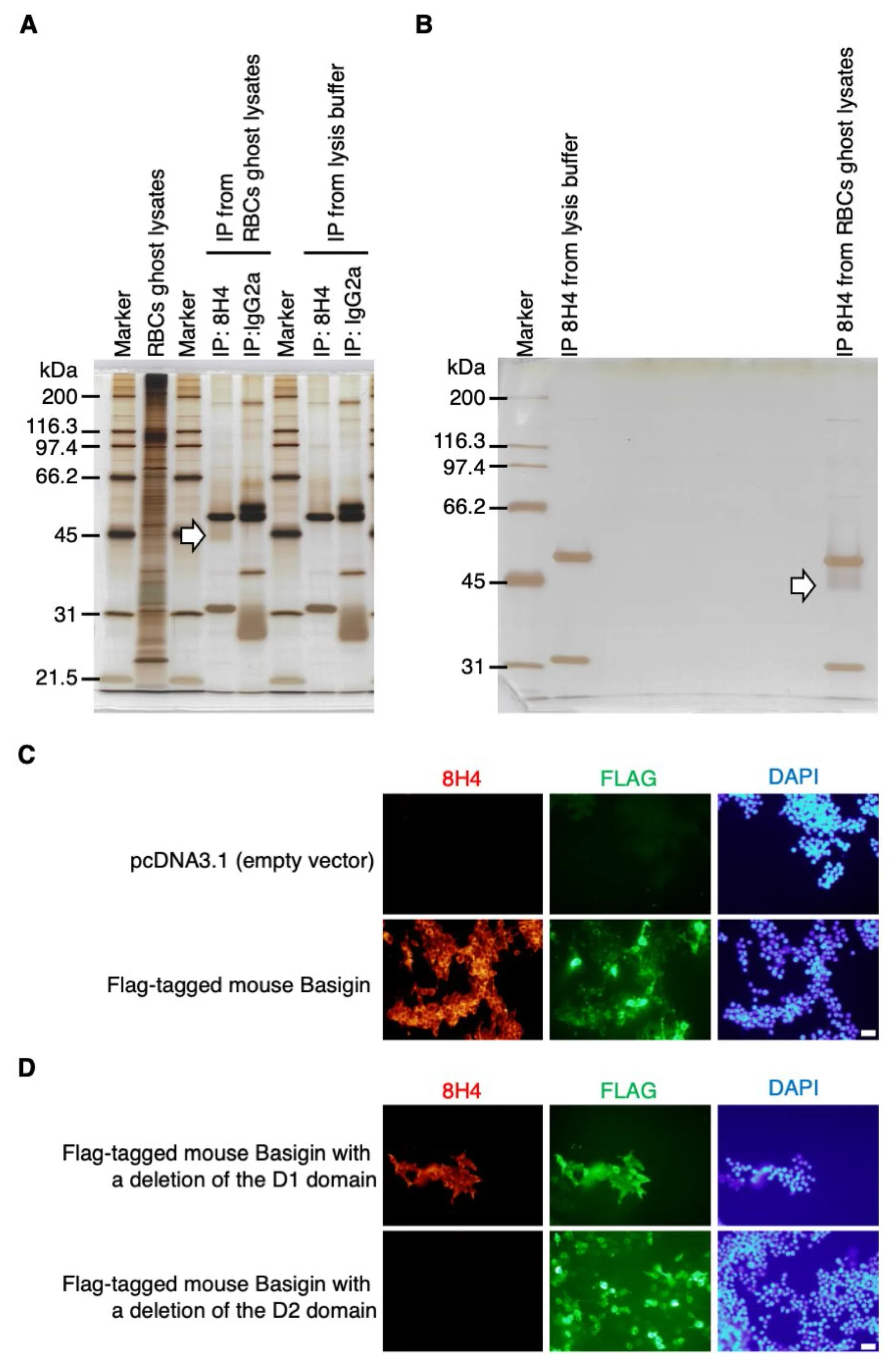
Identification of the antigen recognized by the 8H4 antibody (A) Silver staining of SDS–PAGE gels of RBC ghost lysates and immunoprecipitated samples obtained using the indicated antibodies from RBC ghost lysates or lysis buffer. Samples were subjected to SDS–PAGE, followed by silver staining using the Silver Stain II Kit Wako (FUJIFILM Wako). The white arrow indicates an approximately 45-kDa protein immunoprecipitated by the 8H4 antibody. IP, immunoprecipitation. (B) Silver staining of SDS–PAGE gels of immunoprecipitated samples prepared for mass spectrometry analysis from RBC ghost lysates or lysis buffer using the 8H4 antibody. Silver staining was performed using a mass spectrometry compatible kit (the Silver Stain MS Kit, FUJIFILM Wako). The white arrow indicates an approximately 45-kDa protein immunoprecipitated by the 8H4 antibody. IP, immunoprecipitation. (C) Representative immunostaining of HEK293 cells expressing Flag-tagged mouse Basigin (lower panels). HEK293 cells transfected with an empty vector (pcDNA3.1) were used as a negative control (upper panels). Cells were immunostained with the 8H4 antibody (left panels) and an anti-Flag antibody (middle panels), and counterstained with DAPI (right panels). Scale bar, 40 μm. (D) Representative immunostaining of HEK293 cells expressing Flag-tagged mouse Basigin with a deletion of the D1 (upper panels) or D2 domain (lower panels). Cells were immunostained with the 8H4 antibody (left panels) and an anti-Flag antibody (middle panels), and counterstained with DAPI (right panels).

**Figure 3:**
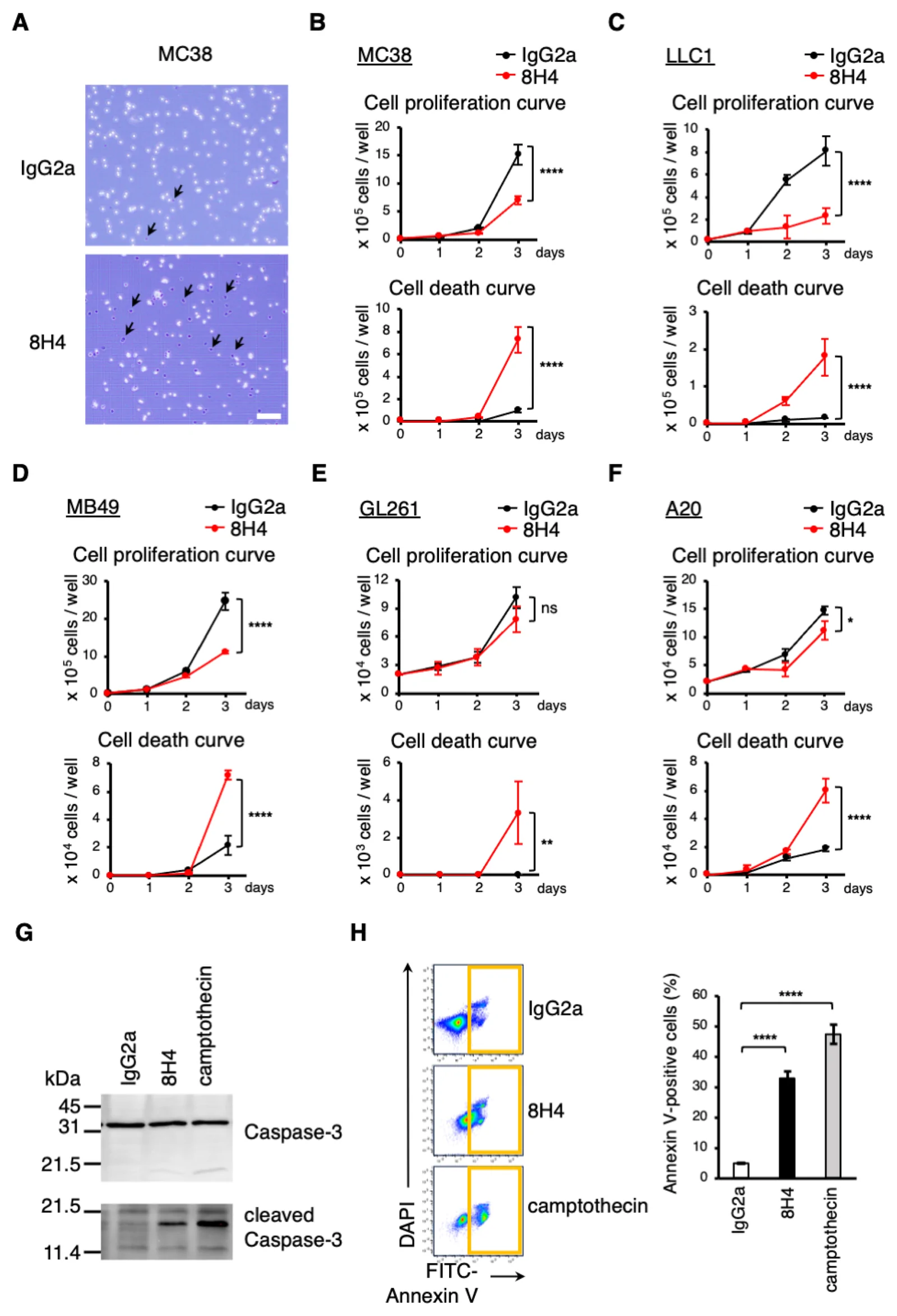
Inhibition of cancer cell growth and survival by the 8H4 antibody (A) Representative images of MC38 cells stained with trypan blue. Arrows indicate trypan blue–positive cells. Scale bar, 200 μm. (B–F) Cell proliferation curves (upper graph) and cell death curves (lower graph) of MC38 (B), LLC1 (C), MB49 (D), GL261 (E), and A20 cells (F) treated with the 8H4 antibody or a control rat IgG2a antibody. Data for cell numbers are means ± SEM (n = 3). (G) Immunoblot analysis with antibodies to cleaved Caspase-3 or to Caspase-3. Camptothecin-treated MC38 cells were included as a positive control for cleaved caspase-3. (H) Representative flow cytometric analysis of MC38 cells treated with the 8H4 antibody or normal rat IgG2a, stained with Annexin V and DAPI (left panels) and the percentage of Annexin V–positive cells among total cells (right panel). Orange squares in the left panels indicate Annexin V–positive MC38 cells. Camptothecin-treated MC38 cells were included as a positive control for apoptosis. Data in the right pannel are means ± SEM (n = 3). *P < 0.05, **P < 0.01, ****P < 0.0001 by 2-way repeated-measures ANOVA with Tukey’s multiple-comparison test (B–F) or by 1-way ANOVA with Tukey’s multiple-comparison test (H). ns, not significant.

**Figure 4:**
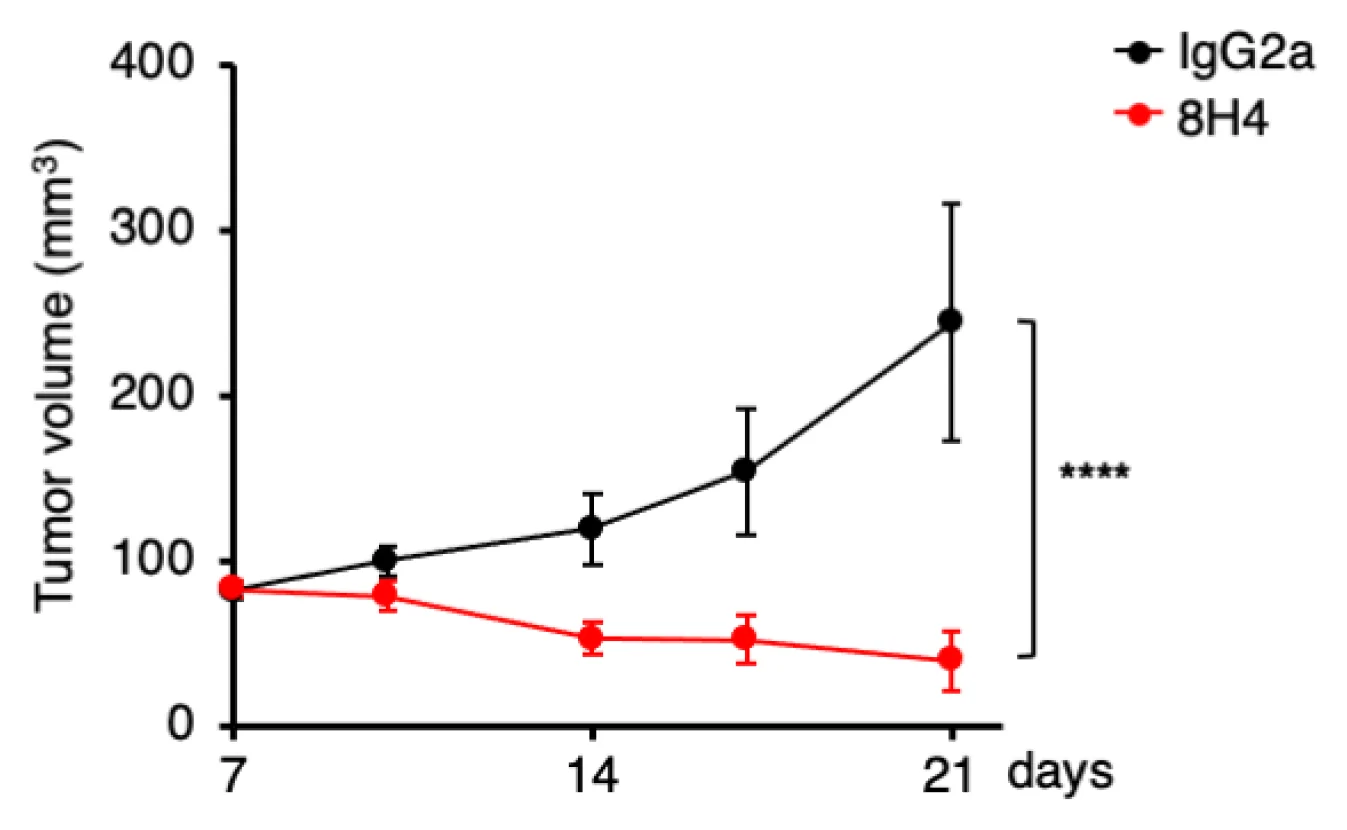
Inhibition of A20 tumor growth by the 8H4 antibody in vivo Tumor volumes for mice injected subcutaneously with A20 cells (5 × 10^6^ cells in 100 μl of PBS). Mice were treated intratumorally with the 8H4 antibody (50 μg/head, n = 15) or normal Rat IgG2a (50 μg/head, n = 15) on days 7, 10, 14, and 17 after A20 cell injection. Data for tumor volume are means ± SEM (n = 15). ****P < 0.0001 by 2-way repeated-measures ANOVA with Tukey’s multiple-comparison test.

**Figure 5:**
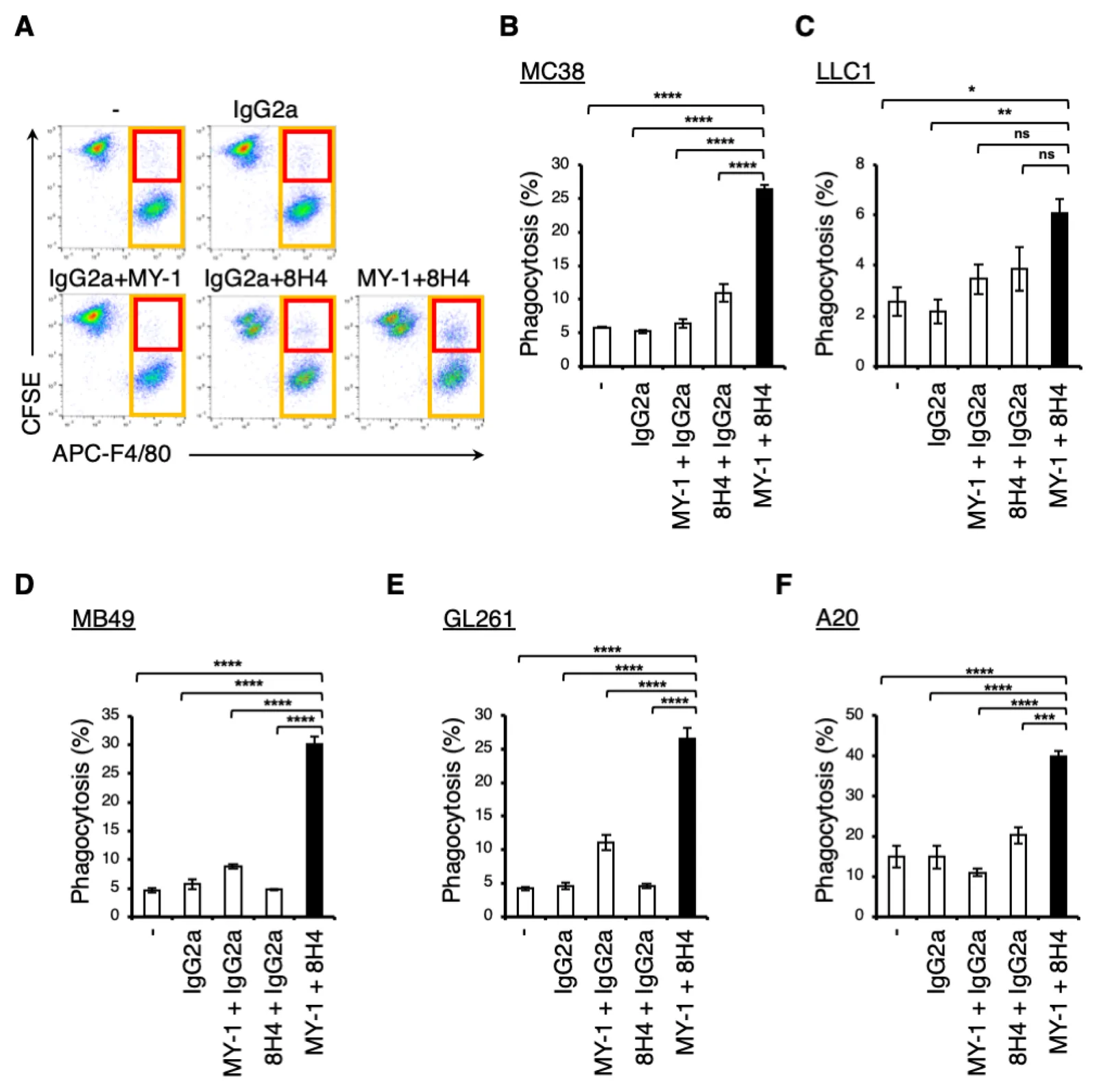
Impact of combination with the 8H4 antibody and an anti-SIRPα antibody (MY-1 antibody) on phagocytosis of cancer cells by macrophages (A) CFSE-labeled MC38 cells (6.0 × 10^4^ cells) were co-incubated with mouse BMDMs (1.5 × 10^4^ cells) in 96-well plates for 4 hours in the presence or absence (−) of the indicated antibodies. Representative flow cytometric plots are shown. Orange squares and red squares indicate total F4/80^+^ BMDMs and CFSE^+^F4/80^+^ BMDMs, respectively. (B–F) CFSE-labeled MC38 (B), LLC1 (C), MB49 (D), GL261 (E), and A20 cells (F) were co-incubated with mouse BMDMs as described in (A) in the presence or absence (−) of the indicated antibodies. The frequency of CFSE^+^F4/80^+^ BMDMs, corresponding to macrophages that had taken up CFSE-labeled cancer cells, was determined as a percentage of the total F4/80^+^ BMDM population. Data are means ± SEM (n = 3). *P < 0.05, **P < 0.01, ***P < 0.001, ****P < 0.0001 by 1-way repeated-measures ANOVA with Tukey’s multiple-comparison test. ns, not significant.
